# Rapid Detection of Carbendazim Residue in Apple Using Surface-Enhanced Raman Scattering and Coupled Chemometric Algorithm

**DOI:** 10.3390/foods11091287

**Published:** 2022-04-28

**Authors:** Xiaowei Huang, Ning Zhang, Zhihua Li, Jiyong Shi, Haroon Elrasheid Tahir, Yue Sun, Yang Zhang, Xinai Zhang, Melvin Holmes, Xiaobo Zou

**Affiliations:** 1 School of Agricultural Engineering, Jiangsu University, Zhenjiang 212013, China; huangxiaowei@ujs.edu.cn (X.H.); zhangning980409@163.com (N.Z.); shi_jiyong@ujs.edu.cn (J.S.); haroona28@yahoo.com (H.E.T.); 15236251775@163.com (Y.S.); yangzhang1@ujs.edu.cn (Y.Z.); zhangxinai@mail.ujs.edu.cn (X.Z.); zou_xiaobo@ujs.edu.cn (X.Z.); 2 School of Food and Biological Engineering, Jiangsu University, Zhenjiang 212013, China; 3 School of Food Science and Nutrition, University of Leeds, Leeds LS2 9JT, UK; 4 International Joint Research Laboratory of Intelligent Agriculture and Agri-Products Processing, Jiangsu Education Department, Jiangsu University, Zhenjiang 212013, China; 5 Collaborative Innovation Center for Modern Grain Circulation and Safety, College of Food Science and Engineering, Nanjing University of Finance and Economics, Nanjing 210023, China

**Keywords:** apple, carbendazim, surface-enhanced Raman spectroscopy (SERS), Ag-NPs @PAN-nanohump arrays, bootstrapping soft shrinkage-partial least squares (BOSS-PLS)

## Abstract

In order to achieve rapid and precise quantification detection of carbendazim residues, surface-enhanced Raman spectroscopy (SERS) combined with variable selected regression methods were developed. A higher sensitivity and greater density of “hot spots” in three-dimensional (3D) SERS substrates based on silver nanoparticles compound polyacrylonitrile (Ag-NPs @PAN) nanohump arrays were fabricated to capture and amplify the SERS signal of carbendazim. Four Raman spectral variable selection regression models were established and comparatively assessed. The results showed that the bootstrapping soft shrinkage-partial least squares (BOSS-PLS) method achieved the best predictive capacity after variable selection, and the final BOSS-PLS model has the correlation coefficient (*R*_P_) of 0.992. Then, this method used to detect the carbendazim residue in apple samples; the recoveries were 86~116%, and relative standard deviation (RSD) is less than 10%. The 3D SERS substrates combined with the BOSS-PLS algorithm can deliver a simple and accurate method for trace detection of carbendazim residues in apples.

## 1. Introduction

Apples are one of the most consumed fruits globally due to their widespread cultivation and extensive range of cultivars, which provide significant consumer choice. However, due to increased awareness of food safety, supplying high-quality and safe fruit is of great importance for the apple producers and the retailers [1]. However, apple crops can be exposed to many diseases during the maturation phase, which requires the regular application of pesticides and fungicides [2]. Among all the pesticides applied, carbendazim is widely used in apple cultivation as a broad-spectrum bactericidal for prevention and treatment of fungal diseases [3,4]. However, excessive use and consumption of carbendazim residues induce toxic effects in humans and other species. Due to the differences in regional climates and diet, acceptable levels of pesticide residues vary worldwide [5,6]. Assessing appropriate pesticide usage and the potential dietary intake risk requires significant analysis and modelling to define acceptable application limits and concentration within fruit samples. In China, the maximum standard limit for carbendazim residues in apple is 5 mg/kg [7]. In order to reduce the exposure risk of carbendazim, it is essential to qualitatively monitor and quantitatively detect residues on agricultural products and in derived products to control exposure levels. Carbendazim levels are commonly determined by analytical techniques, such as gas chromatography (GC), high-performance liquid chromatography (HPLC), gas chromatography-mass spectrometer (GC–MS) [8], liquid chromatography-mass spectrometer (LC-MS) [9,10], and enzyme-linked immunosorbent assay (ELISA) [11]. These methods can achieve high reproducibility and accuracy but have inherent limitations, such as being time-consuming and requiring expensive instrumentation and skilled operators. Thus, it is necessary to establish more simple, rapid, and accurate analytical methods for the analyses of microelements or trace pesticide residues.

Surface-enhanced Raman scattering (SERS) technology can enhance Raman spectroscopy signals and rapidly obtain target molecular spectral fingerprint information with high reproducibility with the advantage of simple sample preparation. Accordingly, SERS is also a technique used to detect trace substances, especially pesticide residues [12]. Au, Ag, and other precious metals contain a large number of free electrons and can produce surface plasmon resonance phenomenon. The plasma on the rough metal surface has resonance effect under the excitation of incident light, which increases the intensity of the Raman signal. Among them, Ag is more widely used because of its relatively stable properties and low price. Furini et al. [13] fabricated Ag NPs with about 50 nm spherical shape. These Ag-NPs effectively enhance the Raman signal for the selective detection of carbendazim at 1228 cm^−1^ characteristic peak. He et al. [14] proposed a method to increase the number of hot spots though 3D silver microspheres (Ag MSs) nanoparticle junctions. The method detected the concentration of carbendazim within the linear range of 0.1–10 mg/L with a detection limit of 0.01 mg/L. With the widespread application of pesticides and commercialization and the necessity for systematic monitoring of residue levels to ensure food safety, there is also substantial use of spectrometry as a rapid analytical technique. However, in their use, the potential measurement errors introduce the possibility of inaccuracies, which compromise food safety and potentially loss of production through rejection of produce. Therefore, an enhanced system to address these issues would have obvious benefits. The introduction and usage of an enhanced SERS system can be a suitable solution to the problem of rapid pesticide residue detection. However, organic matter composition complexity of agricultural products greatly increases the interference of the Raman spectral signal [15,16]. How to extract the characteristic signal and eliminate the interference is the key challenge [17].

**Figure 1:**
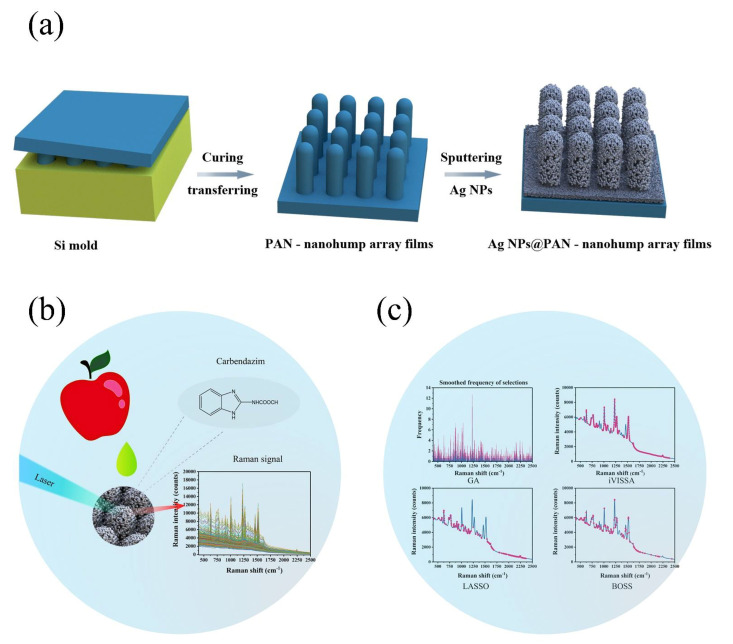
(**a**) Scheme of the fabrication of the SERS chip; (**b**) schematic representation of the detection of carbendazim residues in apple; (**c**) schematic illustration the chemometric algorithms.

In this study, the use of chemometrics was employed in conjunction with a novel sensor. As shown in Figure 1, higher sensitivity and greater density of “hot spots” within a three-dimensional (3D) SERS substrate was fabricated through a regular Ag-sputtering method and applied for carbendazim Raman signal capture. Because of the additional vertical coupling effects by the high-density 3D SERS active hot spot, the Raman signals from the Ag-nanoparticle (Ag-NP)-decorated polyacrylonitrile (PAN) nanohump (denoted as Ag-NPs @PAN-nanohump) arrays substrates were stronger than those from one-layer Ag-NPs. In order to eliminate the interference signal, the variable selection algorithm was applied to extract characteristics spectral before the carbendazim prediction model establishment. This research provides a rapid quantitative detection model of carbendazim residue in apple.

## 2. Materials and Methods

### 2.1. Reagents and Materials

Polyacrylonitrile (PAN, Mw 150,000) was purchased from Sigma-Aldrich. Dimethyl formamide (DMF), ethanol, acetonitrile, 4-Mercaptophenol, and carbendazim were purchased from Shanghai Aladdin Reagent Co., Ltd. (Shanghai, China). Anhydrous MgSO_4_ and NaCl were purchased from Sinopharm Chemical Reagent Co., Ltd. (Shanghai, China). The water used in all experiments was ultrapure (18.2 MΩ). All chemicals were used as received without further purification. Fuji apple samples were brought from the local farm market, Zhenjiang, Jiangsu. The purchased Fuji apples grow in four different geographical origins: Xinjiang province, Shandong province, Shanxi province, and Gansu province.

### 2.2. Preparation of Ag-NPs @PAN-Nanohump Array Film

Ag-NP @PAN-nanohump array film was prepared according to a previous study [18]. Briefly, firstly, a Polyacrylonitrile (PAN) solution was prepared by dissolving 8 wt% of PAN powder in DMF. Then, 250 μL of as-prepared PAN solution was cast onto the well-designed Si mold uniformly. The mold was heated at 70 °C for 20 min to evaporate the solvent. Then a PAN-nanohump array film was demolded and directly transferred onto an appropriate substrate (e.g., Si wafers, glass). Before sputtering the Ag-NPs onto the surfaces of the PAN-nanohump array film via regular Ag-sputtering method, the as-prepared PAN-nanohump array film were fixed on flat Si wafers, which were fixed on the rotatable stage of the sputter coater (EMITECH K550X sputter coater), and a galvanic current of sputtering 40 mA was applied with sputtering duration of 20 min at an interval of 2 min with a vacuum of 0.1 mBar at room temperature. The topography of Ag-NPs @PAN-nanohump array film were characterized by scanning electron microscope (SEM, Quanta FEG 250, FEI, Hillsboro, OR, USA). The nanostructures elemental composition of the Ag-NPs @PAN-nanohump were analyzed by the energy dispersive spectroscopy (EDS, Quanta FEG 250, FEI, Hillsboro, OR, USA).

### 2.3. SERS Measurement

A standard stock carbendazim solution (50 mg/L) was prepared by dissolving carbendazim powder in ethanol/water solvent (ethanol:H_2_O = 1:1), and then, a 50 mg/L stock solution was diluted with ethanol/water solution to different concentrations (0.1, 0.5, 1, 5, 10, 20, and 50 mg/L). An ethanol/water solvent (ethanol: H_2_O = 1:1) without carbendazim was used as control sample. Then different concentrations of carbendazim and the blank control were deposited into the chip with optimal Ag-NPs @PAN-nanohump array film for the quantitative testing. The 36 spectra were collected for each concentration by Confocal Micro-Raman imaging spectrometer (XploRA Plus, HORIBA, Pairs, France). Excitation wavelength was 785 nm; acquisition time was set as 1 s at 1 accumulation and the filter was set as 25%; parameters of the objective lens are 10×. The spectrum range of all the spectrum is 400 to 2500 cm^−1^. Data were collected with a high enhancement factor (EF) about of 1.1 × 10^7^ (Figure S1) to ensure the acquisition of SERS spectra.

### 2.4. Preparation of Apple Samples

The homogenized apple sample (10 g) and 10 mL acetonitrile were mixed in a 50 mL centrifuge tube with the dispenser, and the sample was agitated vigorously for 1min by using oscillator at maximum speed. Then, 4 g anhydrous MgSO_4_ and 1 g NaCl was added and mixed on an oscillator immediately for 1 min. Next, the carbendazim standard solution was added [19]. The mixture was oscillated for another 30 s and centrifuged for 5 min at 5000 rpm. The concentrations of the standard solutions were 0.1, 0.5, 1, 5, 10, 20, and 50 mg/L. Afterwards, the centrifugalized supernatant was filtered through a 0.22 μm membrane syringe filter. Finally, the filtrates were combined for SERS analysis.

### 2.5. HPLC-MS Measurement

HPLC-MS method was used as a standard method to determine the amount of carbendazim in apple (Thermo LXQ LC/MS, Thermo Fisher Scientific, Waltham, MA, USA). The mobile phase was 70% ammonium acetate and 30% acetonitrile (*v*/*v*). The elution speed was 0.3 mL/min, and column temperature was maintained at 25 °C [7]. For the MS analyses, the capillary voltage was 3.6 kV. The desolvation temperature and gas flow was 500 °C and 800 L/h, respectively. Argon (99.99%) was used as the collision gas with a pressure of 2 × 10^−3^ mbar in the T-wave cell [3].

### 2.6. Specificity and Selectivity of the Ag-NPs @PAN-Nanohump-Array Film

Five other pesticides (chlorpyrifos, thiram, parathion-methyl, captan, and isocarbophos) were selected for an interference test to assess the selectivity and specificity of this method. The interfering pesticides were prepared at the concentration of 10 mg/L with ethanol/water (1:1, *v*/*v*) solution as solvent. Under the same detection conditions, the concentration of carbendazim was 0.01 mg/L.

### 2.7. Data Analysis

Raw Raman spectra contained baseline shift, background information, stochastic noise, and sample information. In this study, a multiplicative scatter correction (MSC) preprocessing method was used to eliminate the interferences in the original Raman spectrum before modeling. Partial least squares (PLS) regression is widely used for developing linear models in spectral analysis. This method is an effective multivariate statistical regression technique, but the full spectrum contains many thousands of variables [20]. Amongst these, there are many “uninformative variables” that are not associated with the carbendazim composition and their interaction under measurement. Therefore, variable selection methods were combined with PLS to screen useful variables and so reduce the final PLS factors required to specify the solution [21]. In this study, a Genetic Algorithm (GA), interval Variable Iterative Space Shrinkage Approach (iVISSA), Least Absolute Shrinkage and Selection Operator (LASSO), and Bootstrapping Soft Shrinkage (BOSS) were used for selection of optimal variables. The optimal variables were employed for the subsequent modeling process. All data analyses and algorithms were implemented in MATLAB R2016a and Origin 2017.

## 3. Results and Discussion

### 3.1. Characterization of the Prepared Ag-NPs @PAN-Nanohump Array Film

**Figure 2:**
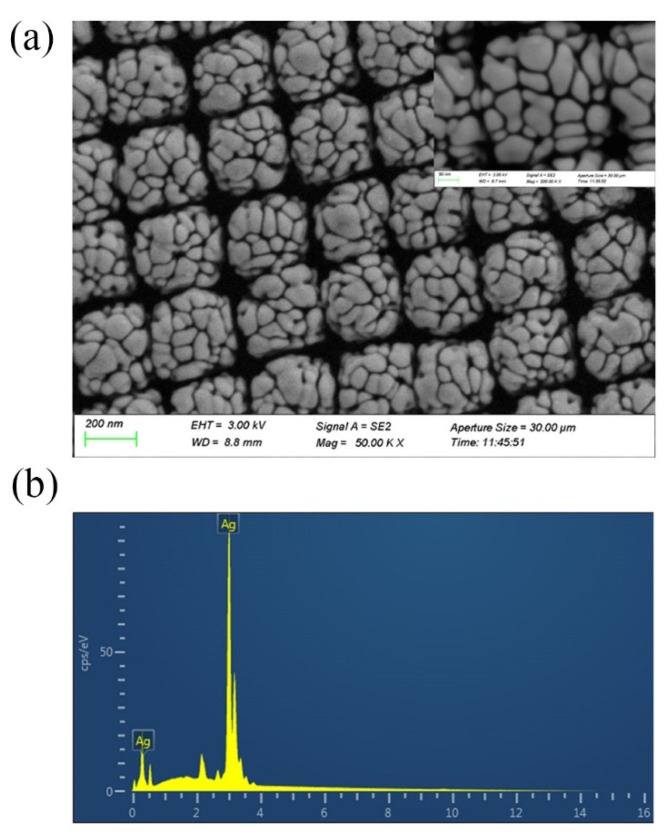
(**a**) SEM images of Ag-NPs @PAN-nano-hemisphere film. (**b**) The existence of Ag in the EDS pattern confirmed the elemental composition of Ag-NPs @PAN-nano-hemisphere film.

The SEM images of Ag-NPs @PAN-nanohump array film are shown in Figure 2a. The molded structures reveal highly ordered tetragonal arrays of nano-hemispheres with diameters of approximately 250 nm. The images also indicate that silver nanoparticles with sphere-like and rod-like morphology compactly clustered on the surface of PAN-nano-hemisphere array; this provides high-density “hot spots” in their gaps to enhance the Raman signals. The Figure 2b shown the existence of Ag in the EDS pattern confirmed the elemental composition of Ag-NPs @PAN-nanohump array film.

### 3.2. Determination of Carbendazim with Ag-NPs @PAN-Nanohump Array Film

**Figure 3:**
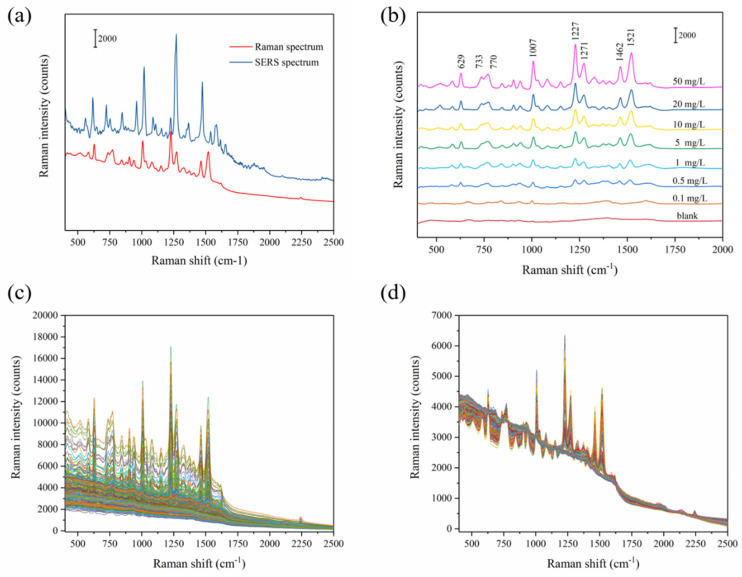
Raman spectrum and SERS spectrum of carbendazim (**a**); raw SERS spectra with seven different concentrations of carbendazim (**b**); 252 SERS spectra of carbendazim (**c**); and the MSC preprocessed spectra (**d**).

The Original Raman spectra of carbendazim powder and SERS spectrum of 50 carbendazim mg/L are shown in Figure 3a. The SERS spectra of carbendazim at different concentrations (range from 0.1 to 50 mg/L) after being adsorbed on Ag-NPs @PAN-nanohump array film are shown in Figure 3b. Even at low concentrations (0.1 mg/L), the characteristic carbendazim peaks were observed. The peak position of Raman spectrum appears at 629, 733, 770, 1007, 1227, 1271, 1462, and 1521 cm^−1^, which were consistent with the Raman characteristic peak of carbendazim shown in previous studies [13]. The bands assigned to the benzimidazole group localized at 629 cm^−1^, 733 cm^−1^, 1227 cm^−1^, and 1521 cm^−1^ are clearly seen. The results suggested that the benzimidazole group interacts with the SERS chip surface, thereby being closer to the surface nanoparticle than the aliphatic group. Additionally, compared with Raman spectroscopy in Table 1, the SERS peaks of carbendazim showed a shift deviation. The different concentrations from 0.01 to 50 mg/L (0.1, 0.5, 1, 5, 10, 20, and 50 mg/L) were introduced to the Ag-NPs @PAN-nanohump array film for SERS carbendazim quantitative measurements, shown in Figure 3c. A total of 252 spectra of carbendazim standard solutions with the concentrations from 400 to 2500 cm^−1^ (1416 wavenumbers) were collected.

In order to eliminate interference information in raw spectrum, an appropriate spectrum pretreatment method was selected. Multiplicative scatter correction (MSC) can remove artifacts or imperfections (e.g., undesirable scatter effect) from the data matrix before data modeling by means of separating the chemical light absorption from the physical light scatter [22]. Therefore, MSC was adopted as an acceptable spectrum pre-processing method in this study. The MSC pretreated Raman spectra were shown in Figure 3d. Then, all 252 samples were randomly divided into 2:1 calibration and prediction set for establishing and verifying models, respectively. The grouping principle is as follows: all samples are (randomly arranged) sorted according to y value (i.e., the carbendazim reference value) from high to low. After 2/1 division, one is selected randomly from every three spectra for the prediction set to avoid bias in subset division.

### 3.3. Model Results

The performance of the model was assessed according to the root mean square error of cross-validation (RMSECV) and correlation coefficient value (*R*_C_) of calibration set, root mean squared error on prediction (RMSEP), and correlation coefficient value of the prediction set (*R*_P_). The higher *R*_P_ and *R*_C_ values indicate better precise predictability and higher correlativity, and the lower RMSEP and RMSECV values imply fine stability of model. In addition, for quantitative models, the ratio of prediction to deviation value (RPD) is the ratio of the standard deviation (SD) to RMSEP. It is generally considered that for the RPD greater than two, the model can be used for quantitative prediction [23].

The four variable selection methods used in this study have different principles and characteristics. GA simulates the natural selection evolutionary process genetics of Darwin’s biological evolution theory to search the optimal solution [24]. GA has the advantages of flexible search and good global search. However, it was chosen also because of the randomness of variable selection inference; a large amount of computations run multiple times is required to avoid error under the same conditions. Therefore, as spectral intensities are measured at a very large number of wavelengths, the search domain increases correspondingly, and the detection of the relevant regions is much more difficult [25]. iVISSA searches the locations and combinations of informative wavelengths in the global search procedure, whereas it determines the widths of wavelength intervals according to the information of continuity in spectroscopic data in the local search procedure [26,27,28]. LASSO has a wide range of applications with low data requirements and can handle both continuous and discrete dependent variables [29]. It can also filter variables and reduce the complexity of the model. The LASSO method tends to compress the coefficients with large absolute values to an excessive degree, which results in model deviation [30,31]. For the BOSS algorithm, the regression coefficient information is used in a specified and propitious way termed “soft shrinkage” [32]. The soft shrinkage strategy assigns smaller weights to the less informative variables, so it has the opportunity to participate in the sub-model for further judgment [33,34]. By comparing these four models, RPD values of all models were greater than three, which mean these models could be used to quantitatively predict the carbendazim based on Raman spectrum. The variables selected from the full spectra and prediction model were shown in Figure S2. According to the *R*_P_, *R*_C_ values and RMSEP, RMSECV values, the BOSS-PLS showed the best prediction performance compared to the other three models in Table 2. Not only that, but the BOSS was applied to screen and select Raman spectrum variables with significant information relevant to carbendazim even with collinearity. Through these results, it is shown that Raman spectra technique combined with BOSS-PLS model to quantify carbendazim obtained the most satisfactory results.

### 3.4. Specificity and Selectivity

**Figure 4:**
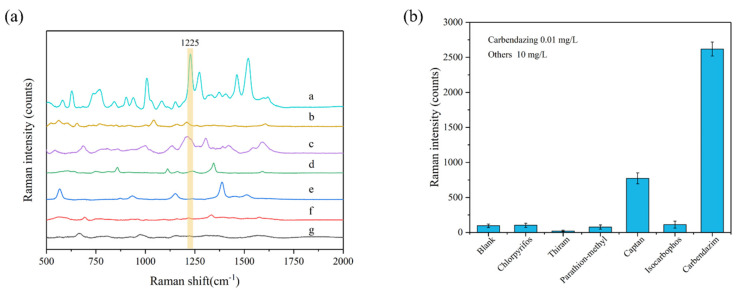
(**a**) SERS spectra and (**b**) peak intensity at 1225 cm^−1^ of carbendazim (0.01 mg/L) and other five pesticides (10 mg/L) in ethanol/water (1:1, *v*/*v*) solution. The spectra from a to g are carbendazim, isocarbophos, captan, parathion-methyl, thiram, chlorpyrifos, and blank samples.

To assess the specificity and selectivity of this method, the Raman signals of five other pesticides, including chlorpyrifos, thiram, parathion-methyl, captan, and isocarbophos, were measured under the same conditions. The results of this interference factors test are shown in Figure 4. The peak at 1225 cm^−1^ can be used as the characteristic peak of carbendazim to achieve specific detection; at this characteristic peak, the signal strength of all five interfering pesticides were much lower than that of carbendazim. Therefore, the Ag-NPs @PAN-nanohump array film substrates presented outstanding specificity and selectivity for detecting carbendazim at 1225 cm^−1^ characteristic Raman peak. In addition, the prepared 3D NPs are also of good stability after two weeks, as shown in Figure S3.

### 3.5. Detection of Carbendazim in Apple Samples

Apple samples were spiked with five different concentrations of carbendazim (0.1, 0.5, 1.0, 5.0, and 10.0 mg/L), and 50 replicated samples were prepared. The 50 samples were assigned as a test set to verify the BOSS-PLS model prediction performance. The standard addition recovery rate of the carbendazim detected by Ag-NPs @PAN-nanohump array film was 86~116%, and the relative standard deviations were 1.68~3.54%, as depicted in Table S1. The results showed that Ag-NPs @PAN-nanohump array film was a simple and precise method to rapidly detect carbendazim in real samples and met the requirements of national standards.

### 3.6. Comparison with LC-MS Methods

In order to demonstrate the reliability of the method, the results of Ag-NPs @PAN-nanohump array film combined with the BOSS-PLS model detection in actual samples were compared with HPLC-MS results (Figure S4). The HPLC-MS chromatogram and standard curve of carbendazim standard solution are shown in Figure S4a,b respectively. The retention time of carbendazim in HPLC-MS chromatogram is 1.57 min. The correlation coefficient (*R*^2^) of carbendazim standard solution is 0.9994. Carbendazim standard addition methods were used here, and the standard addition recovery rate of the carbendazim detected by HPLC-MS was 89~106%, and the relative standard deviations were 1.57~3.52%, as depicted in Table 3. Compared with SERS, the standard addition recovery rate of the carbendazim detected by HPLC-MS was much closer to 100%. The HPLC-MS detection results also show smaller random error and higher precision. However, Ag-NPs @PAN-nanohump array film combined with BOSS-PLS model has high speed and cheap costs and accurate testing, and it is also more suitable for online, large sample size detection and so offers superior operational capabilities.

Then, to further verify the practicability of Ag-NPs @PAN-nanohump array film, carbendazim concentrations of 50 spiked apple samples were measured by SERS combined with the BOSS-PLS method as the predicted values and the results of HPLC-MS method set as the measured values. Table 3 shows the comparison results between SERS and HPLC-MS method for the detection of carbendazim in apple samples. The relative error values are within a range of ±10%, and the *R*_C_ = 0.9874 of BOSS-PLS method. Compared to HPLC-MS, the SERS method is easier and more rapid to implement. SERS combined with the BOSS-PLS method can provide satisfactory qualitative and quantitative results comparable to that of HPLC-MS.

## 4. Conclusions

This study proposed SERS technology combined with a chemometric model to detect carbendazim residues in apples. Ag-NPs @PAN-nanohump array 3D SERS substrate with high EF was fabricated to collect the SERS spectrum of carbendazim at varying concentrations. Spectral pretreatment, variable selection, and PLS algorithms were used to process SERS spectral data and construct carbendazim prediction models. The Ag-NPs @PAN-nanohump array film substrates presented excellent specificity and selectivity for carbendazim. The established BOSS-PLS model applied to MSC pretreated calibration set and prediction data set showed the best prediction performance. The method provides a rapid and sensitive method to detect trace pesticide residues and so improve food quality and safety assurance.

## Figures and Tables

**Table 1:** The band assignments of major peaks for carbendazim SERS spectra.

Assignment	This Work		Reported	
	Raman (cm^−1^) (Carbendazim Solid Samples)	SERS (cm^−1^) (Carbendazim with the SERS Substrates)	SERS Wavenumbers	References
Ring stretching C-C bending	617	629	628	[13,14]
C-H bending in benzene ring	722	736	733	[14]
C-H wagging	751	770	774	[14]
C-N bending C-C stretch C-O-CH_3_ stretching	1018	1007	1007	[13,14]
C-C stretch C-H bending N-H bending	1225	1227	1228	[13,14]
C-H bending N-H bending	1268	1271	1277	[13]
N-H bending C-H bending	1473	1462	1460	[13,14]
N-H bending C-N stretch	1538	1521	1523	[13,14]

**Table 2:** The results of different models applied to select Raman spectrum variables and develop prediction model of carbendazim.

Variable Selection Regression Model	Principal Components	Selected Variables	Selected Wavelength cm^−1^	Calibration Set		Prediction Set		RPD
				*R*_C_	RMSEC (mg/L)	*R*_P_	RMSEP (mg/L)	
iVISSA-PLS	8	945	1227, 1272, and 1543	0.9864	0.334	0.9828	0.358	3.355
GA-PLS	8	99	629, 1007, 1227, 1271, 1521	0.9854	0.340	0.9857	0.331	3.370
LASSO-PLS	10	218	629, 736, and 770	0.9885	0.295	0.9890	0.312	4.112
BOSS-PLS	8	136	629, 736, 770, 1007, 1227, 1271, 1462, and 1521	0.9898	0.286	0.9923	0.247	4.169

**Table 3:** Comparison between SERS with BOSS-PLS model and LC-MS/MS method for the detection of carbendazim in apple.

Carbendazim Added (mg/kg)	SERS with BOSS-PLS Model (mg/kg)	Recovery Rate (%)	RSD (%) *n* = 10	LC-MS/MS (mg/kg)	Recovery Rate (%)	RSD (%) *n* = 10	BOSS-PLS Prediction Model
0	0 ± 0.013	0	0	0	0	0	
0.1	0.101 ± 0.015	86~116	3.54	0.097 ± 0.008	89~106	3.52	*R*_C_ = 0.987 RMSEC = 0.241 (mg/kg) *R*_P_ = 0.973 RMSEP = 0.314 (mg/kg)
0.5	0.498 ± 0.011		2.15	0.501 ± 0.022		1.57	
1.0	1.022 ± 0.033		1.74	1.031 ± 0.028		2.01	
5.0	5.141 ± 0.078		2.14	5.087 ± 0.084		1.59	
10.0	10.315 ± 0.374		1.68	10.227 ± 0.214		1.97	

## Data Availability

Data is contained within the article.

## Supplementary Materials

The following supporting information can be downloaded at: https://www.mdpi.com/article/10.3390/foods11091287/s1, Figure S1. (a) SERS spectrum of 2.5 μL of 1 × 10^−6^ M 4-Mercaphenol ethanol solution was dispersed to an area of 36 mm^2^ for the Ag-NPs @PAN-nano-hump array film. (b). Raman spectrum of 2.5 μL of 1 × 10^−3^ M 4-Mercaphenol ethanol solution was dispersed to an area of 20 mm^2^ for the silicon wafer. The exposure time was 60 s. Figure S2. (a) the frequency of variable selection after 100 runs by GA; (b) GA-PLS model result; (c) the distributions of selected variables for the iVISSA-PLS built model; (d) iVISSA-PLS model performance results; (e) spectral variables selected by LASSO algorithm based on preprocessed spectra of Carbendazim; (f) LASSO-PLS model performance results. (g) the distributions of selected variables for the BOSS-PLS built model; (h) BOSS-PLS model results. Figure S3. The stability of the system. Figure S4. The chromatogram of carbendazim standard solution (a). the relationship between peak area and the concentration of carbendazim standard solution (b). Chromatographic conditions: mobile phase, Ammonium acetate. Table S1. model results by PLS of the original data and spectral data pretreated with MSC.
