# Supplementary material for: Rapid Detection of Carbendazim Residue in Apple Using Surface-Enhanced Raman Scattering and Coupled Chemometric Algorithm
Source: Foods. 2022 Apr 28;11(9):1287. doi: 10.3390/foods11091287 (PMC9103909; doi:10.3390/foods11091287)
Supplement: Supplementary file 1 [file foods-11-01287-s001.zip › foods-1672818-supplementary.pdf]

Supplementary material

Rapid Detection of Carbendazim Residue in Apple Using Surface-Enhanced Raman Scattering and Coupled Chemometric Algorithm

Huang Xiaowei [1], Zhang Ning [1], Li Zhihua [1,*], Shi Jiyong [1], Haroon Elrasheid Tahir [1], Sun Yue [1], Zhang Yang [1], Zhang Xinai [1], Melvin Holmes [2,3,*] and Zou Xiaobo [1,3,4]

[1] School of Agricultural Engineering, School of Food and Biological Engineering, Jiangsu University, 301 Xuefu Rd., Zhenjiang, 212013, China; huangxiaowei@ujs.edu.cn (H.X.); zhangning980409@163.com (Z.N.); shi_jiyong@ujs.edu.cn (S.J.); haroona28@yahoo.com (H.E.T.); 15236251775@163.com (S.Y.); yangzhang1@ujs.edu.cn (Z.Y.); zhangxinai@mail.ujs.edu.cn (Z.X.); zou_xiaobo@ujs.edu.cn (Z.X.)

[2] School of Food Science and Nutrition, University of Leeds, Leeds LS2 9JT, UK

[3] International Joint Research Laboratory of Intelligent Agriculture and Agri-Products Processing, Jiangsu Education Department, Jiangsu University, Zhenjiang, 212013, China

[4] Collaborative Innovation Center for Modern Grain Circulation and Safety, College of Food Science and Engineering, Nanjing University of Finance and Economics, 128 North Railway Street, Gulou District, Nanjing 210023, China

* Correspondence: lizh@ujs.edu.cn (L.Z.); prcmjh@leeds.ac.uk (M.H.); Tel.: +86-511-88780085 (L.Z.);
Fax: +86-511-88780201 (L.Z.)

Estimation of enhancement factor

To examine the enhancement factor (EF) of prepared SERS substrates, 4-Mercaphenol commonly used SERS molecules were measured. The SERS EF is a quantitative measure of the Raman signal amplification of the analyte. The EF value using the reported protocol. The EF can be calculated by:

$$EF = \frac{I_{SERS}N_{Ref}}{I_{Ref}N_{SERS}} \qquad \text{(S1)}$$

Where N_{SERS} and N_{Ref} are the number of molecules probed on the Ag-NPs @PAN-nanohump array film and on the reference sample, respectively. I_{SERS} and I_{Ref} correspond to SERS signal and un-enhanced normal signals intensities, respectively. Herein, a certain volume (V_{SERS}) and concentration (C_{SERS}) 4-Mercaptophenol ethanol solution was dispersed to an area of S_{SERS} at the Ag-NPs @PAN-nanohump array film substrate. For non-SERS Raman spectra, a certain volume (V_{Ref}) and concentration (C_{Ref}) 4-Mercaphenol ethanol solution was dispersed to an area of S_{Ref} at a clean Si substrate. Both the substrates were dried in the air. Considering the area of laser spot is the same, the foregoing equation thus becomes:

$$EF = \frac{I_{SERS}}{I_{Ref}} \bullet \frac{C_{Ref}V_{Ref}}{C_{SERS}V_{SERS}} \bullet \frac{S_{SERS}}{S_{Ref}} \qquad \text{(S2)}$$

In this experiment, 2.5μL of 1×10^{-9}M 4-Mercaphenol ethanol solution was dispersed to an area of 36mm^2 for the Ag-NPs @PAN-nanohump array film substrate and 2.5μL of 1×10^{-3}M 4-

Mercaphenol ethanol solution was dispersed to an area of 20mm² for the silicon wafer. For the band at 1078cm⁻¹, ISERS/IRef was 11107/1815=6.12 There is average enhancement factor for the band at 1078cm-1 is calculated to be 1.1×10^{7}.

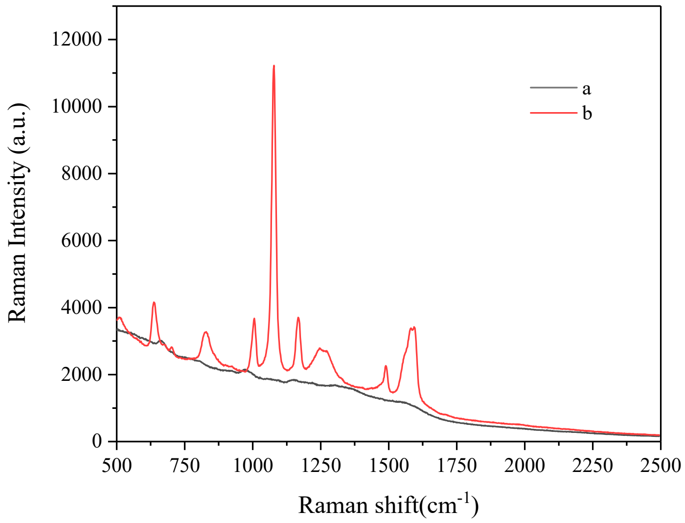

Figure S1. (a) SERS spectrum of 2.5μL of 1×10^{-6} M 4-Mercaphenol ethanol solution was dispersed to an area of 36 mm² for the Ag-NPs @PAN-nano-hump array film. (b). Raman spectrum of 2.5μL of 1×10^{-3}M 4-Mercaphenol ethanol solution was dispersed to an area of 20mm² for the silicon wafer. The exposure time was 60s.

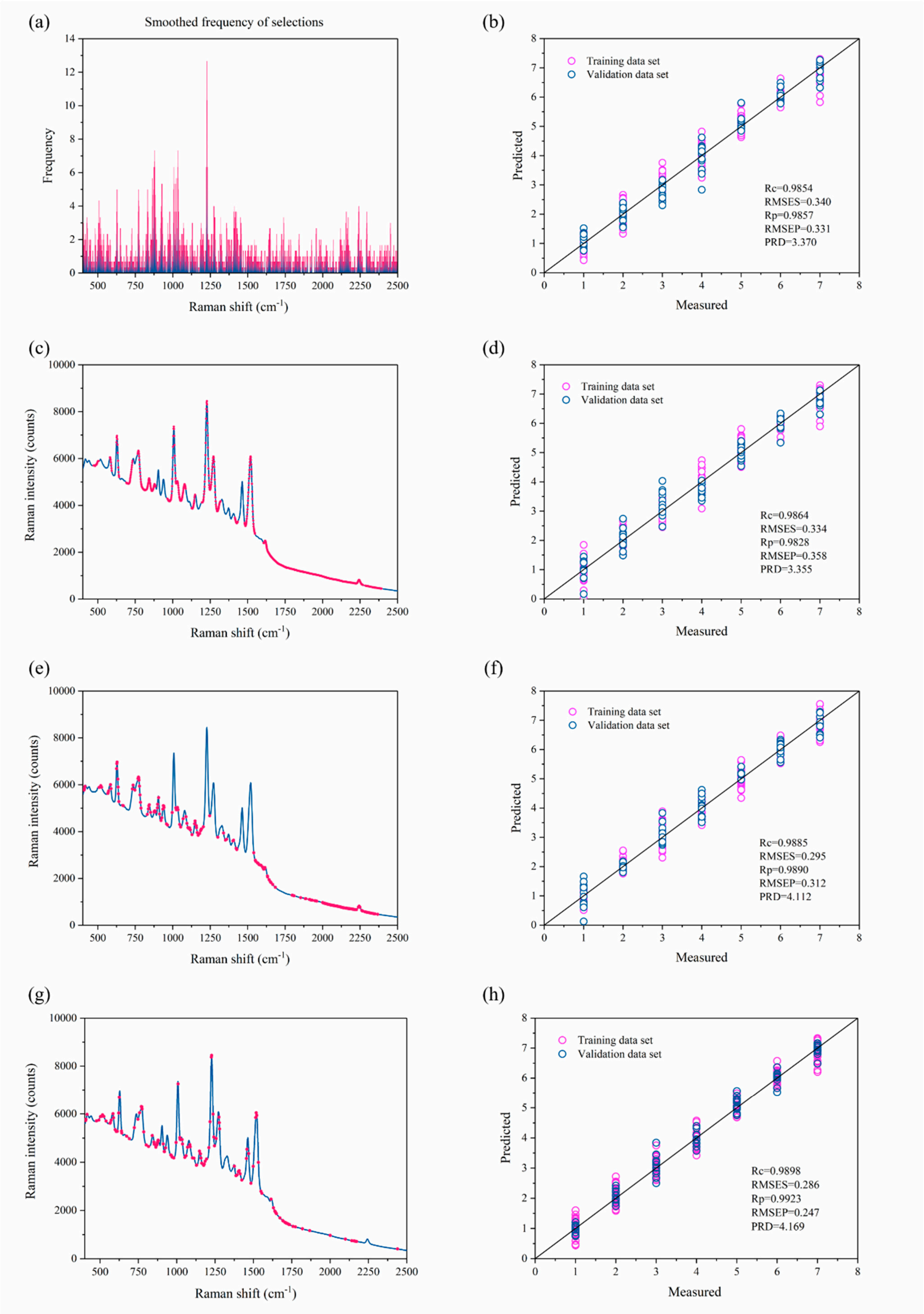
(a)
Smoothed frequency of selections
Frequency
Raman shift (cm-1)
(b)
Training data set
Validation data set
Predicted
Measured
Rc=0.9854
RMSES=0.340
Rp=0.9857
RMSEP=0.331
PRD=3.370
(c)
Raman intensity (counts)
Raman shift (cm-1)
(d)
Training data set
Validation data set
Predicted
Measured
Rc=0.9864
RMSES=0.334
Rp=0.9828
RMSEP=0.358
PRD=3.355
(e)
Raman intensity (counts)
Raman shift (cm-1)
(f)
Training data set
Validation data set
Predicted
Measured
Rc=0.9885
RMSES=0.295
Rp=0.9890
RMSEP=0.312
PRD=4.112
(g)
Raman intensity (counts)
Raman shift (cm-1)
(h)
Training data set
Validation data set
Predicted
Measured
Rc=0.9898
RMSES=0.286
Rp=0.9923
RMSEP=0.247
PRD=4.169

Figure S2. (a) the frequency of variable selection after 100 runs by GA; (b) GA-PLS model result ;(c) the distributions of selected variables for the iVISSA-PLS built model; (d)iVISSA-PLS model performance results; (e) spectral variables selected by LASSO algorithm based on preprocessed spectra of Carbendazim; (f) LASSO-PLS model performance results. (g) the distributions of selected variables for the BOSS-PLS built model; (h) BOSS-PLS model results.

The stability of the system

To examine stability of the method, Ag-NPs @PAN-nanohump array film stored for a certain period was used to detect the carbendazim at concentrations of 0.1, 0.5, 1, 5, 10 mg L^{-1}. As displayed in Fig. S3 the detection results of Ag-NPs @PAN-nano-hump array film after different storage days (0, 1, 3, 5, 7, 14 d) remained unchanged, which proved that the prepared Ag-NPs @PAN-nanohump array film has good stability.

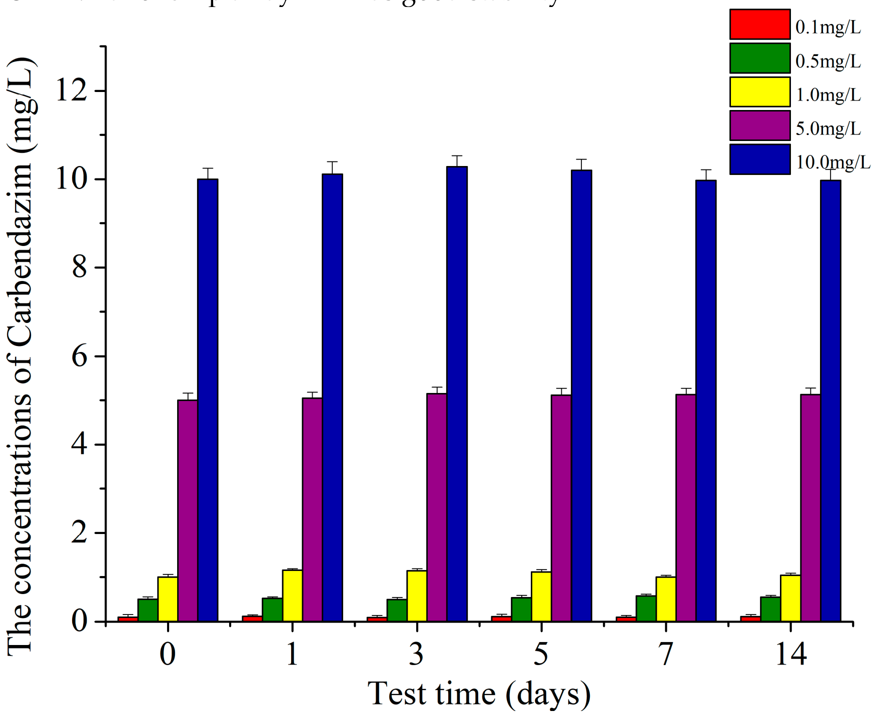

Figure S3. The stability of the system.

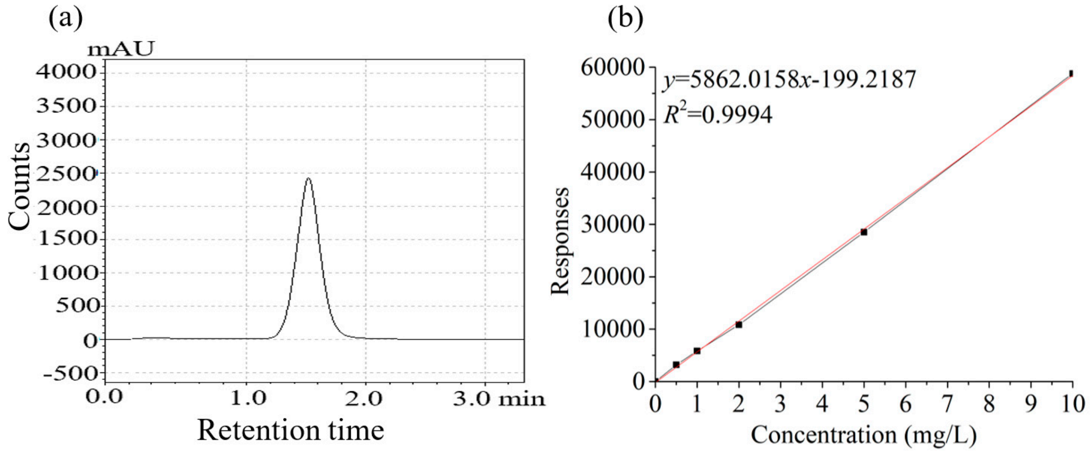

Figure S4. The chromatogram of carbendazim standard solution (a). the relationship between peak area and the concentration of carbendazim standard solution (b). Chromatographic conditions: mobile phase, Ammonium acetate.

Table S1. model results by PLS of the original data and spectral data pretreated with MSC.

Pretreatment methods	Principal components	Variables	Calibration set		Prediction set		RPD
			*R*c	*RMSEC*(mg/L)	*R*p	*RMSEP*(mg/L)	
Original data	8	1416	0.9452	0.626	0.9683	0.505	2.741
MSC	8	1416	0.9854	0.35	0.9829	0.346	3.710
